# Molecular Mechanisms Underlying Autophagy-Mediated Treatment Resistance in Cancer

**DOI:** 10.3390/cancers11111775

**Published:** 2019-11-11

**Authors:** Cally J. Ho, Sharon M. Gorski

**Affiliations:** 1 Canada’s Michael Smith Genome Sciences Centre, BC Cancer, Vancouver, BC V5Z 1L3, Canada; caho@bcgsc.ca; 2 Department of Molecular Biology and Biochemistry, Simon Fraser University, Burnaby, BC V5A 1S6, Canada; 3 Centre for Cell Biology, Development, and Disease, Simon Fraser University, Burnaby, BC V5A 1S6, Canada

**Keywords:** autophagy, cancer, treatment resistance, targeted agents, chemotherapy, molecular mechanisms, chemoresistance

## Abstract

Despite advances in diagnostic tools and therapeutic options, treatment resistance remains a challenge for many cancer patients. Recent studies have found evidence that autophagy, a cellular pathway that delivers cytoplasmic components to lysosomes for degradation and recycling, contributes to treatment resistance in different cancer types. A role for autophagy in resistance to chemotherapies and targeted therapies has been described based largely on associations with various signaling pathways, including MAPK and PI3K/AKT signaling. However, our current understanding of the molecular mechanisms underlying the role of autophagy in facilitating treatment resistance remains limited. Here we provide a comprehensive summary of the evidence linking autophagy to major signaling pathways in the context of treatment resistance and tumor progression, and then highlight recently emerged molecular mechanisms underlying autophagy and the p62/KEAP1/NRF2 and FOXO3A/PUMA axes in chemoresistance.

## 1. Introduction

Autophagy is an intracellular degradative pathway that delivers cytoplasmic components to lysosomes for degradation and recycling. The term “autophagy” is derived from the Greek words “auto” meaning oneself and “phagy” meaning to eat and was first coined by Christian de Duve at the 1963 Ciba Foundation Symposium on Lysosomes. In mammalian systems, there are at least three co-existing forms of autophagy that are morphologically distinct, as follows: Microautophagy, chaperone-mediated autophagy (CMA), and macroautophagy [1,2]. Microautophagy is characterized by the uptake of small cytoplasmic fragments into lysosomes through the formation of inward lysosomal membrane invaginations. This is unlike CMA, where chaperone proteins facilitate the direct uptake and translocation of cytosolic components into lysosomes for degradation and recycling [1,2]. Macroautophagy is characterized by the formation of double-membrane structures, known as autophagosomes, that fuse with lysosomes to form autolysosomes that degrade and recycle engulfed cellular components [3,4]. Macroautophagy is the most extensively studied form of autophagy and is the main mechanism used by eukaryotes for the maintenance of cellular homeostasis and quality control [3,4].

Significant progress has been made over the past decade in regards to our understanding of the roles of macroautophagy (hereafter referred to as autophagy) in health and disease [5,6]. In particular, autophagy has been shown to play both pro- and anti-tumorigenic roles during the onset and progression of cancers, and in response to anti-cancer treatment [7,8]. Autophagy functions in tumor suppression during early stages of tumorigenesis by maintaining cellular homeostasis and genome stability through the clearance of cytotoxic proteins and damaged organelles, and by the regulation of cell death and senescence [9,10,11,12,13]. During later stages of cancer progression, autophagy favors tumorigenesis by contributing to tumor survival under conditions of oxidative stress and nutrient deprivation, by initiating cellular survival responses and catabolizing redundant organelles and proteins for energy [14,15,16,17,18,19,20]. Recent excellent reviews cover the tumor-promoting and -suppressive roles of autophagy in cancer in greater detail [7,21,22]. The pro-tumorigenic roles of autophagy have primed it as an attractive therapeutic target for cancer treatments [23,24,25]. Autophagy can be modulated through genetic approaches, like small interfering RNAs (siRNAs) and small hairpin RNAs (shRNAs) that target key autophagy-related (ATG) genes. Many pharmacological compounds that inhibit different stages of autophagy have also been developed and have been used to inhibit autophagy (Table 1). Despite many ongoing preclinical and clinical studies investigating the therapeutic benefit of autophagy inhibition alone or in combination treatment strategies in cancers [26,27,28], our current understanding of the actual molecular mechanisms underlying the pro-tumorigenic contributions of autophagy to treatment resistance remains largely unknown.

## 2. Autophagy Contributes to Treatment Resistance in Cancer

Tumor initiation is largely stochastic by nature and involves a coordinated destabilization of major cellular processes. The dynamic and evolutionary manner by which this occurs creates molecularly heterogenous tumors [29,30]. The ability of cancers to adapt to and survive the effects of cancer therapies remains one of the greatest impediments in medical and clinical oncology. Treatment resistance directly translates to the ineffectiveness and eventual failures of cancer therapies [31,32,33,34,35,36]. Innate treatment resistance predates therapeutic intervention, whereas acquired treatment resistance is a refractory outcome of cancer therapy that occurs when subpopulations of cancer cells within tumors acquire mutations and adaptations that desensitize them to ongoing treatment [37,38,39,40,41]. To date, treatment resistance remains a major challenge to successful cancer treatment and control, but the mechanisms involved remain poorly understood [42,43].

### 2.1. Autophagy and Resistance Against Chemotherapy

Chemotherapy, with or without surgery and/or radiation, is commonly administered as part of routine first-line treatment of most cancers [44,45]. Chemotherapy involves the use of toxic chemical compounds that target and kill rapidly growing and dividing cells. Most chemotherapeutic agents interfere with the ability of the cells to divide, and often work at the DNA level. Examples include anti-mitotic agents like paclitaxel and docetaxel, topoisomerase II inhibitors (anthracyclines), like doxorubicin and epirubicin, and DNA alkylating agents, such as cisplatin and carboplatin [44,45]. Although such chemotherapeutic agents are systemic and affect normal cells as well, most cancers are characterized by rapid growth and this makes them most amenable to the cytotoxic effects of chemotherapy. However, the therapeutic success of chemotherapy is limited by a large variety of cellular adaptations that provide tumor cells with the ability to tolerate the cytotoxic effects of chemotherapy [45]. Of note, the activation of autophagy in response to standard chemotherapy has been shown to aid in chemoresistance in certain cancer contexts. In such cases, autophagy inhibition in combination with chemotherapy can significantly augment tumor cell killing, supporting a pro-survival role of autophagy in contributing to tumor resistance against chemotherapies (Table 2).

**Table 1:** Examples of pharmacological autophagy inhibitors.

Name	Mechanism of Action	Targeted Stage in Autophagy	Ref.
Inhibitors that target late stages of autophagy			
Lys05	Lysosomotropic agent	Lysosomal acidification Autophagosome-lysosome fusion	[46,47]
Chloroquine (CQ)	Lysosomotropic agent	Lysosomal acidification Autophagosome-lysosome fusion	[48,49]
Hydroxychloroquine (HCQ)			
Quinacrine (QNX)			
IITZ-01	Lysosomotropic agent	Lysosomal acidification Autophagosome-lysosome fusion	[50]
ROC-325	unknown	Lysosomal acidification Autophagosome-lysosome fusion	[51,52,53]
Bafilomycin A1 (Baf A1)	Vacuolar ATPase inhibitor	Lysosomal acidification Autophagosome-lysosome fusion	[54]
ECDD-S27	Vacuolar ATPase inhibitor	Lysosomal acidification Autophagosome-lysosome fusion	[55]
Ca-5f	Unknown	Autophagosome-lysosome fusion	[56]
EACC	Syntaxin 17 (STX17) translocation inhibitor	Autophagosome-lysosome fusion	[57]
MHY1485	Mammalian target of rapamycin (mTOR) activator	Autophagosome-lysosome fusion	[58]
Pepstatin A	Acid protease inhibitor	Lysosomal proteolysis	[59,60]
E64-d	Acid protease inhibitor	Lysosomal proteolysis	[59,60]
Alpha-hederin	Unknown	Lysosomal proteolysis	[61]
Inhibitors that target early stages of autophagy			
SB02024	Vacuolar protein sorting protein 34 (Vps34) inhibitor	Autophagosome formation	[62]
SAR405	Vacuolar protein sorting protein 18 and 34 (Vps18 and Vps34) inhibitor	Autophagosome formation	[63]
PIK-III	Vacuolar protein sorting protein 34 (Vps34) inhibitor	Autophagosome formation	[64]
Autophinib	Vacuolar protein sorting protein 34 (Vps34) inhibitor	Autophagosome formation	[65]
SBI-0206965	Unc-51-like kinase 1 (ULK1) inhibitor	Autophagosome formation	[66]
ULK-100, ULK-101	Unc-51-like kinase 1 (ULK1) inhibitor	Autophagosome formation	[67]
LY294002	Phosphoinositide 3-kinase (PI3) inhibitor	Autophagosome formation	[68,69]
3-Methyladenine (3-MA)	Phosphoinositide 3-kinase (PI3) inhibitor	Autophagosome formation	[70]
Wortmannin	Phosphoinositide 3-kinase (PI3) inhibitor	Autophagosome formation	[68,71,72]
Spautin-1	Ubiquitin Specific Peptidase 10 and 13 (USP10 and USP13) inhibitor	Autophagosome formation	[73,74]
NSC185058	Autophagy-related (ATG) protein 4A and 4B (ATG4A and ATG4B) inhibitor	LC3B, GABARAPL2 priming Autophagosome formation	[75]
UAMC-2526	Autophagy-related (ATG) protein 4B (ATG4B) inhibitor	LC3B priming Autophagosome formation	[76]
DMP-1	unknown	LC3B lipidation Autophagosome formation	[77]
Inhibitors that target both early and late stages of autophagy			
Tioconazole	Autophagy-related (ATG) protein 4A and 4B (ATG4A and ATG4B) inhibitor	LC3B, GABARAPL2 priming LC3B delipidation Autophagosome-lysosome fusion	[78]
LV-320	Autophagy-related (ATG) protein 4A and 4B (ATG4A and ATG4B) inhibitor	LC3B priming LC3B delipidation Autophagosome-lysosome fusion	[79]
S130	Autophagy-related (ATG) protein 4A and 4B (ATG4A and ATG4B) inhibitor	LC3B, GABARAPL2 priming LC3B delipidation	[80]
Xanthium strumarium Fruit Extract	Autophagy-related (ATG) protein 4B (ATG4B) inhibitor	LC3B, GABARAPL2 priming LC3B delipidation	[81]
Verteporfin	unknown	Autophagosome formation Autolysosome degradation	[82]

**Table 2:** Examples of pre-clinical studies demonstrating that autophagy and autophagy-related (ATG) genes contribute to chemotherapy resistance in different types of cancers.

Cancer Type	Chemotherapeutic Agent	Mode of Autophagy Inhibition		Ref.
		Pharmacological	Genetic	
Bladder cancers	Cisplatin, Gemcitabine, Mitomycin, Pirarubicin	CQ, HCQ, Baf A1, Wortmannin, 3-MA	ATG7 shRNA, ATG12 shRNA, BECN1 shRNA, BECN1 siRNA, ATG3 siRNA	[83,84,85,86,87]
Bone cancers	Doxorubicin, Cisplatin, Methotrexate, Paclitaxel	Spautin-1, 3-MA, CQ	BECN1 shRNA, BECN1-targeting deoxyribozyme, miR-410 (ATG16L knockdown), ATG14 siRNA, ATG7 siRNA	[88,89,90,91,92,93,94,95,96,97,98]
Breast cancers	5-Fluorouracil, Doxorubicin, Docetaxel, Adriamycin, Cyclophosphamide, Epirubicin, Paclitaxel, Cisplatin, Gemcitabine	Baf A1, CQ, HCQ, 3-MA, Verteporfin	ATG5 shRNA, ATG5 siRNA, ATG7 siRNA, FIP200 shRNA, ATG13 shRNA, BECN1 shRNA, BECN1 siRNA, BECN1 CRISPR/Cas9 KO, BNIP3L CRISPR/Cas9 KO, AMBRA1 shRNA	[99,100,101,102,103,104,105,106,107]
Cervical cancers	Cisplatin, Oncothermia, Paclitaxel	3-MA, CQ, Baf A1	BECN1 siRNA, ATG5 siRNA, ATG7 siRNA	[108,109,110,111,112]
Colorectal cancers	5-FU, Oxaliplatin	CQ, 3-MA, Baf A1	ATG7 siRNA, BECN1 siRNA, ATG5 siRNA	[113,114,115,116]
Endometrial cancers	Paclitaxel, Cisplatin, Resveratrol, Oncothermia	CQ, 3-MA	BECN1 shRNA, ATG5 siRNA, ATG7 siRNA	[117,118,119,120,121]
Gastric cancers	Vincristine, 5-fluorouracil, Cisplatin, Bufalin, Matrine, Oxaliplatin, Cinobufagin	CQ, 3-MA, Baf A1	miR-23b (ATG12 knockdown), ATG5 siRNA	[122,123,124,125,126,127,128,129,130,131,132]
Gliomas	Temozolomide, Vorinostat	HCQ, CQ, 3-MA, QNX, Baf A1, ATG4B inhibitor (NSC185058)	LC3A siRNA, LC3B siRNA, TFEB siRNA, ATG4C shRNA, ATG4B shRNA	[133,134,135,136,137,138,139,140]
Head and neck cancers	Paclitaxel, Cisplatin, Nedaplatin	3-MA, CQ, Baf A1	LC3B siRNA, ATG3 siRNA, ATG5 siRNA, ATG6 siRNA, ATG7 siRNA	[141,142,143,144,145,146]
Liver cancers	Epirubicin, Oxaliplatin, Mitomycin, Cisplatin, Doxorubicin	3-MA, CQ, Baf A1	ATG4B shRNA, ATG5 siRNA, ATG5 shRNA, ATG7 shRNA, LC3 shRNA, miR-101 (RAB5A, STMN1, ATG4B knockdown)	[147,148,149,150,151,152,153,154,155]
Lung cancers	Paclitaxel, Camptothecin, Cisplatin, 5-fluorouracil, Gemcitabine, Pterostilbene	3-MA, CQ, Alpha-hederin, Baf A1, ATG4B inhibitors (Compound 1, 17)	ATG7 siRNA, BECN1 siRNA, ATG5 siRNA	[61,141,156,157,158,159,160,161,162,163]
Neuroblastomas	Vincristine, Doxorubicin, Cisplatin, Paclitaxel	HCQ, 3-MA	ATG5 shRNA, ATG5 siRNA, BECN1 siRNA, TRP14 siRNA	[164,165,166]
Ovarian cancers	Cisplatin, Carboplatin, Vincristine, Gemcitabine, Ifosfamide, Docetaxel, Paclitaxel	3-MA, CQ, Quinacrine	ATG5 siRNA, BECN1 siRNA, AMBRA1 shRNA, ATG5 CRISPR/Cas9 KO, miR-204 (LC3B and ATG7 knockdown)	[167,168,169,170,171,172,173,174,175,176]
Pancreatic cancers	Doxorubicin, Gemcitabine, Docetaxel	CQ, Verteporfin	ATG7 siRNA, ATG12 siRNA, USP22 siRNA, miR-23b (ATG12 knockdown), miR-29a (ATG9A and TFEB knockdown), miR-29c (USP22 knockdown), miR-137 (ATG5 knockdown)	[177,178,179,180,181,182,183,184,185]
Skin cancers	Temozolomide, Cisplatin	CQ, HCQ, LY294002	ATG5 shRNA	[186,187,188]

Lung cancer is one of the leading causes of cancer-related deaths worldwide, and approximately 80% are defined as non-small cell lung carcinomas (NSCLC) [156]. Treatment regimes administered to NSCLC patients typically include paclitaxel. However, the issue of acquired resistance to paclitaxel in NSCLC remains an obstacle in chemotherapy success [156]. Recent studies have demonstrated that the pharmacological inhibition of autophagy using CQ, prior to paclitaxel treatment, significantly reduces in vitro tumorigenic potential and induces apoptosis of NSCLC cells [156]. Of note, autophagy inhibition also increased intracellular reactive oxidative species (ROS), and this was associated with a reduction in AKT activity [156]. Recent efforts have also been focused on the development of novel small molecule inhibitors of the autophagy related 4B cysteine peptidase 4B (ATG4B), a core cysteine protease in the autophagy process [157]. Pharmacological inhibition of ATG4B was associated with a reduction in autophagic flux, and significantly augmented tumor cell killing effects of tamoxifen or cisplatin in NSCLC cells [157].

Similarly, inhibition of autophagy was shown to improve the responses of osteosarcomas to chemotherapies. Osteosarcoma is a common primary bone cancer that occurs in young adults and, like lung cancer, acquired resistance to chemotherapies remains a clinical challenge [88]. Recent studies have demonstrated that cisplatin-resistant osteosarcoma cells exhibit elevated levels of autophagy, and the pharmacological inhibition of autophagy, using 3-MA, significantly improved sensitivity to cisplatin [88]. Autophagy inhibition was found to increase levels of the FOXO3A transcription factor, which consequently increased levels of the pro-apoptotic protein, PUMA, thereby increasing apoptosis [88]. Interestingly, a similar mechanism of autophagy in treatment resistance was also recently reported and investigated in greater depth in colorectal cancers [189]. We refer our readers to Section 3 of this review for a greater discussion of the findings of this recent paper by Fitzwalter et al. [189]. Taken together, these studies strongly support the role of autophagy in treatment resistance against various chemotherapies in different cancers. Table 2 below provides a more comprehensive summary of examples from pre-clinical studies that support a role for autophagy or autophagy-related (ATG) genes in chemotherapy resistance in a variety of cancer types.

### 2.2. Autophagy and Resistance Against Targeted Agents

Advances in cellular and molecular biology and technology have paved the way for significant improvements in our knowledge of tumor biology, molecular profiling of tumors, and discovery of predictive biomarkers and molecular therapeutic targets [44,45]. These efforts have driven the evolution of cancer treatments and the rise of targeted agents that act selectively on proteins and/or genes that have been altered as part of the pathogenesis of cancer [44,45]. Two main classes of targeted agents are as follows: (i) Small molecules (usually with an “-ib” suffix), that act on cellular targets and interfere with oncogenic signaling pathways, and (ii) monoclonal antibodies (usually with a “-mab” suffix), that bind and inhibit tumor-specific and/or -associated antigens [44,45]. Although targeted therapy has succeeded in prolonging the survival of cancer patients, long-term therapeutic success, again, is limited by the development of molecular and cellular adaptations that confer treatment resistance [190,191]. Both the upregulation of autophagy in response to targeted agents and elevation of autophagy levels in cancers resistant to targeted agents have been observed in many cancer types (Table 3). There is strong pre-clinical support demonstrating that the inhibition of the autophagy process, through either pharmacological and/or genetic means, potentiates the anti-tumorigenic effects of many targeted therapies (Table 3).

Bevacizumab is a monoclonal antibody that binds to and inhibits the cellular activity of the vascular endothelial growth factor (VEGF), a potent angiogenic factor that stimulates the formation of blood vessels. The role of autophagy in resistance to bevacizumab has been demonstrated in several cancers, including gliomas, colorectal cancers, and liver cancers [113,192,193,194,195,196]. Most recently, it has been demonstrated that treatment of glioblastomas with bevacizumab induces autophagy, and the pharmacological inhibition of autophagy, using HCQ, in combination with bevacizumab resulted in a greater mitigation of cancer cell growth compared to either treatment alone [194]. Similarly, bevacizumab treatment was also associated with an upregulation of autophagy in colorectal cancer cells, and the pharmacological or genetic inhibition of autophagy both improved the growth inhibitory and pro-apoptotic effects of bevacizumab in vitro and in vivo [192].

Autophagy inhibition has also been associated with improved responses to small molecule inhibitors, like sorafenib. Sorafenib is a multi-protein kinase inhibitor that inhibits the activity of serine/threonine and receptor tyrosine kinases, including RAF (Rapidly Accelerated Fibrosarcoma) kinases, the VEGF receptor (VEGFR), and platelet-derived growth factor receptors 2/3 (PDGFR 2/3). Recent studies in hepatocellular carcinoma (HCC) demonstrated that pharmacological or genetic inhibition of autophagy in combination with sorafenib resulted in a significant increase in apoptosis and greater reduction in tumor viability [204]. Interestingly, findings from this study were also the first to show an association between the cell surface adhesion protein CD24, sorafenib resistance, and autophagy. Elevated levels of CD24 were associated with poor HCC patient prognosis and sorafenib resistance, and the knockdown of CD24 both improved sorafenib sensitivity and reduced autophagy level [204]. More recently, pharmacological or genetic inhibition of autophagy also improved responses of desmoid tumors to sorafenib [205]. Taken together, these studies provide strong support for the role of autophagy in tumor tolerance against various targeted therapies in diverse cancers. We refer our readers to Table 3 for a more comprehensive list of examples.

### 2.3. Clinical Trials

To date, the only autophagy inhibitors that have been approved by the FDA and used as part of clinical trials in combination with chemotherapy and targeted therapies are the lysosomotropic agent chloroquine (CQ) and its derivative hydroxychloroquine (HCQ). The use of CQ or HCQ as a single agent in the treatment of cancers has been associated with minimal or lack of therapeutic benefit, and this is likely due, in part, to the absence of a secondary agent or stress stimulus that would otherwise induce or create a dependency on the autophagy pathway. To this end, Phase I/II clinical trials that examine the safety and efficacy of CQ or HCQ in combination with various chemotherapies and targeted therapies are underway (http://clinicaltrials.gov/) [27,309,310]. However, dose-limiting toxicities of CQ and HCQ remain a clinical challenge in some patients, with side effects that include neutropenias, retinopathies, and sepsis [27,311,312].

## 3. Crosstalk between Autophagy and Diverse Signaling Pathways Contributes to Treatment Resistance in Cancer

Although previous studies have provided strong evidence supporting a role of autophagy in tumor tolerance against anti-cancer therapies, the molecular mechanisms underlying this relationship remain poorly understood. Existing evidence in the literature thus far has been primarily correlative and suggests crosstalk between autophagy and various signaling pathways that aids and/or supports tumor treatment resistance [313]. Despite our limited understanding of the molecular mechanisms underlying these interplays, knowledge of autophagy-signaling pathway crosstalk has been successfully leveraged at the preclinical stage and targeted to mitigate cancer growth and progression [313]. 

### 3.1. The Contributions of Autophagy and Major Signaling Pathways to Pro-Survival Responses that Resist Anti-Cancer Therapies—Guilt by Association?

In this section, we first review the mitogen-activated protein kinase (MAPK) and phosphoinositide 3-kinase/ protein kinase B (PI3K/AKT) signaling pathways and their known links to cancer progression and therapy resistance. We then describe the associations between these common cancer-related signaling pathways and the autophagy process, along with corresponding implications for treatment resistance.

#### 3.1.1. Mitogen-Activated Protein Kinase (MAPK) Signaling

##### The Role of MAPK Signaling in Cancer Progression

The MAPK signaling pathway is a convergent signaling axis that is critical for the regulation of homeostatic cell growth, proliferation, and cell fate [314]. There are 3 primary arms of MAPK signaling, as follows: (i) Extracellular signal regulated kinases (ERK) signaling, (ii) c-jun N-terminal kinases (JNK) signaling, and (iii) p38 MAPK signaling [314,315]. Each arm is activated by a host of different extracellular stimuli and regulates various downstream transcriptional targets [314,315]. Mutations in genes and aberrations in protein functions involved in MAPK signaling have been known to promote tumor cell survival, metastasis, and resistance to anticancer therapies [316]. MAPK signaling may be initiated by a variety of different growth factor receptors on the cell surface that respond to extracellular stimuli [316]. Some of these growth factor receptors are proteins that belong to the human epidermal growth factor receptor (HER) family, which include HER1, also known as epidermal growth factor receptor (EGFR), and HER2 [317]. Overexpression of or mutations in EGFR and HER2 have been widely observed in certain cancers, like lung [318] and breast [319], and have been associated with tumorigenic activation of MAPK signaling.

**Figure 1:**
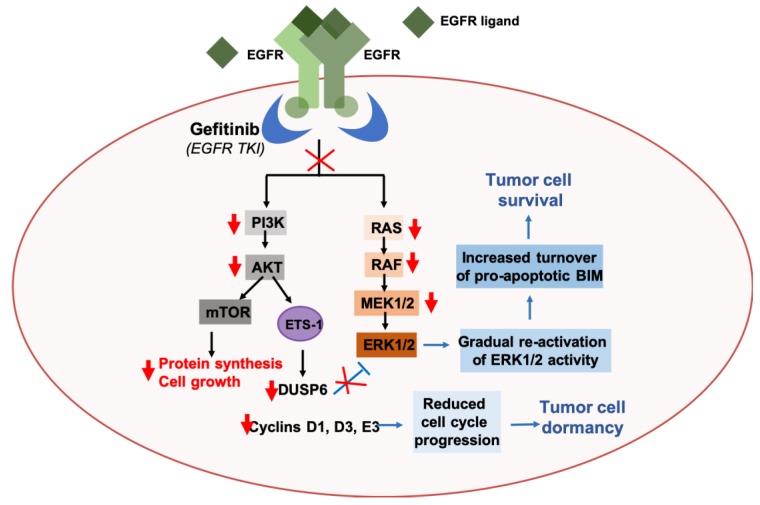
Reactivation of ERK signaling via AKT-ETS-1-mediated positive feedback loop contributes to resistance to the epidermal growth factor receptor (EGFR) tyrosine kinase inhibitor (TKI), Gefitinib, in non-small cell lung cancers (NSCLCs). Gefitinib treatment inhibits EGFR-mediated activation of AKT and ERK1/2 signaling in NSCLCs. Reduced AKT signaling inhibits the activation of the E26 transformation-specific (ETS) transcription factor, Ets-1. Ets-1 is responsible for the transcriptional upregulation of dual specificity phosphatase 6 (DUSP6), a negative regulator of ERK1/2 activity. Reduced DUSP6 levels therefore result in a gradual reactivation of ERK1/2 activity, which promotes the turnover of the pro-apoptotic protein, BIM, and consequently supports tumor cell survival. Reduced Ets-1 activity also leads to a decrease in the transcriptional upregulation of cell cycle regulatory proteins, like cyclins D1, D3, and E3, and this consequently leads to tumor cell dormancy [321].

Approximately 14% of non-small cell lung cancer (NSCLC) patients harbor activating mutations in EGFR, yet do not achieve significant tumor reduction in response to EGFR tyrosine kinase inhibitors (TKIs) [320]. It was previously demonstrated that treatment with EGFR TKIs is associated with gradual re-activation of MAPK signaling in cell line models of NSCLC harboring EGFR mutations, following an initial inhibition of both ERK and protein kinase B (PKB/AKT) signaling [321]. The re-activation of MAPK signaling occurs as a result of a reduction in AKT activity, which blocks the activation of the transcription factor ETS-1. Inhibition of ETS-1 activity reduces levels of the dual specificity phosphatase 6 (DUSP6), a negative regulator of ERK1/2, and this consequently allows for the gradual reactivation of ERK1/2. Overall, this leads to tumor cell dormancy, as a result of reduced expression of ETS-1 target genes that are involved in cell cycle regulation, and survival of NSCLC cells, through sustained ERK1/2 activity and increased turnover of the pro-apoptotic protein, B-cell lymphoma-2-like protein 11 (BIM) (Figure 1) [321]. Results from this study demonstrate that MAPK/ERK signaling plays an important role in cancer cell survival and growth and suggest a novel crosstalk between MAPK and AKT signaling that contributes to innate resistance of NSCLCs to EGFR TKIs. We refer our readers to recent reviews that cover the various other mechanisms of resistance to EGFR TKIs in cancers in greater detail [322,323,324,325]. Clinical trials investigating the potential of targeting MAPK/ERK signaling in NSCLC patients are currently underway, with the recent clinical approval of the combined use of the Raf inhibitor dafarenib, and the MEK inhibitor trametinib, in patients with metastatic NSCLC [326,327].

**Figure 2:**
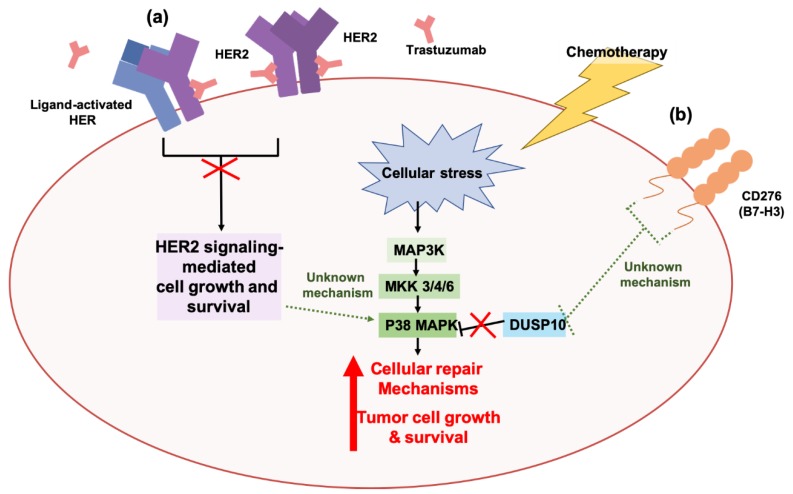
The stress-activated p38 MAPK pathway contributes to tumor resistance against targeted therapies and chemotherapies in cancers. (**a**) Overexpression of the human epidermal growth factor receptor 2 (HER2) protein has been observed in cancers, like breast, and has been associated with the aberrant activation of growth and survival pathways, like AKT signaling and ERK signaling. The HER2-targeted antibody trastuzumab binds to the extracellular domain of HER2 and inhibits HER2 dimerization and HER2-mediated signaling events, consequently mitigating tumorigenesis. Ectopic activation of the p38 MAPK pathway in HER2-overexpressing (HER2+) breast cancers can confer resistance to trastuzumab by promoting cell growth and survival independent of HER2 activity [331]. To date, the mechanisms underlying the relationship between HER2 signaling and p38 MAPK remain unknown. (**b**) Resistance to chemotherapies, like cisplatin and dacarbazine, have also been associated with p38 MAPK activity in melanoma cells. Chemotherapy-induced cellular stress activates the p38 MAPK pathway, and this results in the activation of cellular repair mechanisms that promote tumor cell growth and survival. Activation of p38 MAPK is further sustained by the immune checkpoint molecule, CD276/B7-H3, which inhibits the p38 MAPK-negative regulator dual specificity protein phosphatase 10 (DUSP10) [335]. To date, the mechanistic relationship between CD276/B7-H3 activity and DUSP10 inhibition remains unknown.

Similarly, the HER2 protein is overexpressed in approximately 25% of breast cancer patients [328]. Although patient survival outcomes have significantly improved since the development and approval of the anti-HER2 targeted agent trastuzumab, the issue of treatment resistance still remains a challenge in advanced/metastatic cases of HER2+ breast cancer patients [329,330]. Previous studies have demonstrated that the p38 MAPK facilitates resistance to trastuzumab in breast cancers that overexpress the HER2 protein [331]. Both pharmacological and genetic inhibition of p38 MAPK sensitized trastuzumab-refractory HER2+ breast cancers that exhibit elevated activation of p38 MAPK to trastuzumab [331]. Genetic induction of p38 MAPK activation was also shown to increase cellular invasiveness and confer tolerance to trastuzumab in cells previously sensitive to trastuzumab [331], supporting the role of p38 MAPK signaling in promoting the tumorigenic potential of HER2+ breast cancers and their tolerance to trastuzumab (Figure 2). We refer our readers to recent reviews that cover the various other mechanisms of resistance to trastuzumab in cancers in greater detail [329,332,333,334]. More recently, p38 MAPK was also implicated in treatment resistance against the chemotherapeutic agents cisplatin and dacarbazine, through the immune checkpoint molecule, CD276 (B7-H3), in melanoma cells [335]. High expression of CD276 has been associated with poor patient survival in cancers, like pancreatic cancer [336] and renal cell carcinoma [337]. Genetic inhibition of CD276 increased the sensitivity of melanoma cells to cisplatin and dacarbazine. Increased levels of dual specificity protein phosphatase 10 (DUSP10), a negative regulator of p38 MAPK activity, was also observed, and genetic inhibition of DUSP10 in CD276-knockdown melanoma cells was associated with an increase in p38 MAPK activation and drug resistance. These studies suggest that a novel CD276-DUSP10-p38 MAPK axis functions in the regulation of chemoresistance in melanomas, and supports the role of p38 MAPK in chemoresistance [335] (Figure 2).

Dysregulation of p38 MAPK signaling has also been reported in other cancer types and associated with poor clinical outcomes [338,339]. However, despite their clinical potential, the therapeutic value of current p38 MAPK inhibitors is limited by their high toxicities and off-target effects [340,341]. Clinical trials examining the potential of targeting the p38 MAPK pathway using novel small molecule compounds in advanced cancers are currently underway [342,343] (http://clinicaltrials.gov/).

##### MAPK Signaling and Autophagy: Connections to Treatment Resistance in Cancers

**Figure 3:**
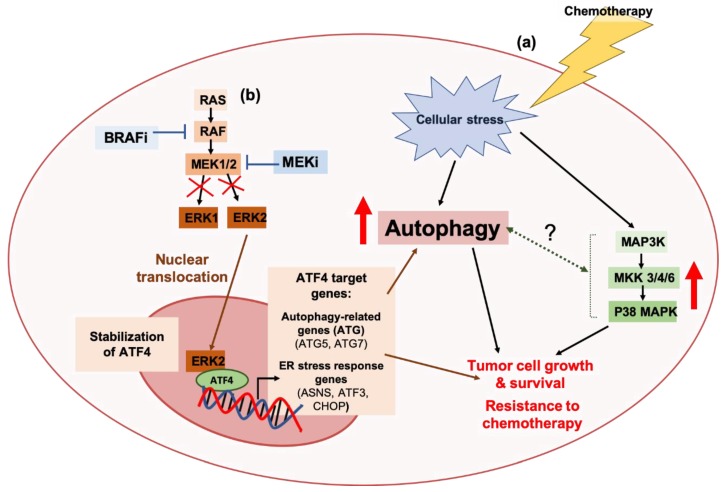
Associations between the autophagy pathway and MAPK signaling in to tumor tolerance against targeted therapies and chemotherapies. (**a**) Chemotherapeutic agents, like 5-fluorouracil, activate the p38 MAPK pathway in response to cellular stress, and thus promote cell survival in colorectal cancers. Pharmacological or genetic inhibition of p38 MAPK induces the autophagy pathway, and autophagy inhibition in p38 MAPK-deficient colorectal cancer cells potentiates the cytotoxic effects of chemotherapy [344]. To date, the relationship between autophagy regulation and the p38 MAPK remains poorly understood. (**b**) Crosstalk between the autophagy pathway and the ERK signaling arm of the MAPK pathway has been associated with resistance to B-RAF inhibitors (BRAFi) or MEK inhibitors (MEKi) in melanomas. BRAFi or MEKi-mediated inhibition of ERK1/2 signaling has been associated with the nuclear translocation of ERK2, and stabilization of the transcription factor, Activating Transcription Factor 4 (ATF4). This promotes the transcriptional upregulation of ATF4 target genes, like autophagy-related genes (ATGs) ATG5 and ATG7, and ER stress response genes, Asparagine synthetase 3 (ASN3), Cyclic AMP-dependent transcription factor (ATF3), and C/EBP-Homologous Protein (CHOP) [345].

Both MAPK signaling and the autophagy pathway have been shown to promote tumor tolerance to anti-cancer therapies. Resistance to the anti-metabolite 5-fluorouracil (5-FU), which is a commonly used chemotherapy in cancers like colorectal cancers, has been linked to increased activation of p38 MAPK [344]. In such cases of chemoresistance, inhibition of p38 MAPK has been associated with elevated autophagy levels, suggesting that autophagy is upregulated under conditions of 5-FU-induced chemotherapeutic stress to compensate for loss of p38 MAPK signaling [344]. It was also demonstrated that subsequent inhibition of autophagy in p38 MAPK-deficient cancer cells was synergistic in increasing sensitivity to 5-FU, suggesting a crosstalk between the autophagy pathway and p38 MAPK signaling that confers resistance to chemotherapy [344] (Figure 3). Associations between the autophagy pathway and MAPK/ERK signaling have also been drawn and implicated in resistance to BRAF inhibitors (BRAFi) and MEK inhibitors (MEKi) in melanoma. Studies have demonstrated that, in response to BRAFi or MEKi, major signaling components of the MAPK/ERK pathway translocate initially to the endoplasmic reticulum (ER) and then to the nucleus to activate autophagy and ER stress responses [345]. Of note, it was shown that translocation of ERK2 to the nucleus aids in the stabilization of the transcription factor Activating Transcription Factor 4 (ATF4), which acts to transcriptionally activate proteins involved in ER stress response and autophagy [345]. It was further shown that patient-derived melanoma xenografts (PDX) that were resistant to combined HCQ, dabrafenib, and trametinib treatments presented with an upregulation of MAPK components in the ER, an increase in nuclear levels of activated ATF4, and an upregulation of autophagy levels when exposed to MEKi or BRAFi [345]. These observations suggest a model whereby crosstalk occurs between the autophagy pathway and MAPK/ERK signaling function to promote resistance to BRAFi and MEKi in melanomas, mediating tumor growth and survival [345] (Figure 3). Most recently, the concurrent inhibition of autophagy and ERK activity in pancreatic ductal adenocarcinomas (PDAC) was also found to be additive in suppressing tumor growth and survival both in vitro and in vivo [287,346], further suggesting a crosstalk between MAPK signaling and autophagy in promoting resistance to anti-cancer treatments [347].

To date, the actual molecular mechanisms underlying how MAPK signaling interacts with the autophagy pathway to promote tumor tolerance against anti-cancer therapies remains unclear. Regardless, at least 3 clinical trials investigating the therapeutic potential of concomitant targeting of MAPK signaling, through trametinib, and the autophagy pathway, through HCQ, in cancers are in progress (http://clinicaltrials.gov/).

#### 3.1.2. Protein Kinase B/AKT (PKB/AKT) Pathway

##### The Role of PKB/AKT Signaling in Cancer Progression

The PKB pathway, hereafter referred to as the AKT pathway, is a major signaling node that also plays important roles in the regulation of various cellular processes [348]. Of the three classes of Phosphoinositide-3-Kinases (PI3Ks), AKT signaling is regulated by the class I. Class II PI3Ks primarily regulate membrane trafficking, whereas class III PI3Ks have been implicated in the regulation of both membrane trafficking and nutrient sensing [348]. Unlike class II and III, class I PI3Ks have demonstrated roles in the regulation of cellular growth, survival, and protein synthesis [348]. Class I PI3Ks (hereafter referred to as PI3Ks) are activated via signals received from activated cell surface receptors, like the G-protein coupled receptors (GPCRs), activated Ras, and the receptor tyrosine kinases EGFR and HER2. Activated PI3Ks mediate the phosphorylation of the phospholipid PtdIns (4,5) P2 to PtdIns(4,5)P3, which functions as a secondary messenger that facilitates the recruitment and activation of kinases that belong to the protein kinase A, G, and C family, like serum/glucocorticoid-regulated kinases (SGKs) and AKT. AKT is a major downstream target of PI3K activation, and is responsible for the subsequent activation of the mammalian target of rapamycin complex 1 (mTORC1), which plays a vital role in the regulation of cellular protein synthesis and survival [348].

**Figure 4:**
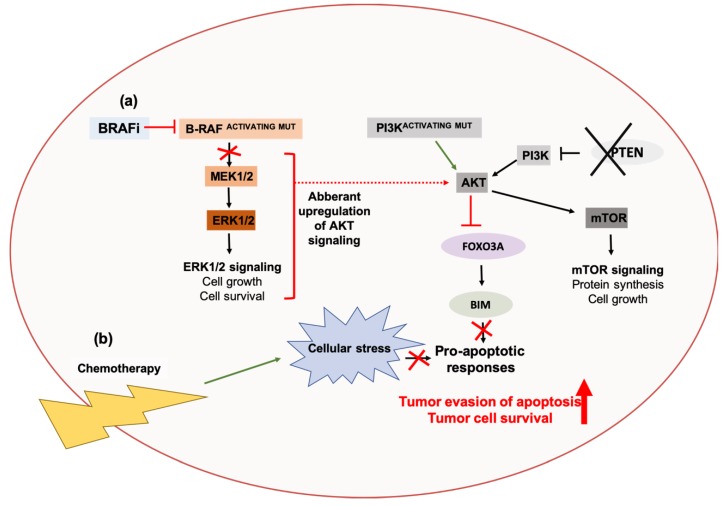
The AKT signaling pathway contributes to treatment resistance against targeted therapies and chemotherapies in cancers. (**a**) BRAF inhibition (BRAFi) upregulates AKT activity in melanomas with concurrent PTEN loss and activating BRAFv600E mutations. This result in AKT-mediated inhibition of the transcription factor Forkhead Box O3A (FOXO3A), and reduces the transcriptional upregulation of its pro-apoptotic target gene, BIM. Combined pharmacological inhibition of BRAF activity and AKT activity, by PI3K inhibition, increases BIM expression and potentiates apoptosis, suggesting that AKT activity contributes to the resistance of BRAFV600E mutation-bearing melanomas to BRAFi [352]. (**b**) Activating mutations in the catalytic subunit of PI3K (PI3KCA) also contribute to resistance against chemotherapies in colorectal cancers through suppression of pro-apoptotic responses, and inhibition of PI3K can potentiate the cytotoxic effects of chemotherapy [353].

Like MAPK signaling, the AKT pathway can also be initiated by various growth factor receptors, including the HER proteins [348]. Aberrations in the regulation of AKT signaling and its pathway components have been implicated in tumorigenesis and the development of resistance to anti-cancer therapies in many cancers [349]. The phosphatase and tensin homolog (PTEN) protein is a key negative regulator of PI3K/AKT signaling, and functions as a phosphatase that inhibits AKT activity via dephosphorylation of PtdIns (4,5) P3. Loss of PTEN function is a frequent observation in many cases of melanoma and has been associated with poor clinical outcomes [350,351]. Concurrently, most patients also present with activating BRAF mutations, but a subset of such patients possess tumors that are insensitive to BRAF inhibitors [350,351]. It was previously demonstrated that PTEN is required for the upregulation of the pro-apoptotic protein, BIM, following inhibition of BRAF. In melanomas that present with concurrent PTEN loss and activating BRAFV600E mutations, BRAF inhibition results in aberrant upregulation of AKT signaling, causing constitutive AKT-mediated inhibition of the transcription factor FOXO3A, and this consequently results in a downregulation of BIM and impairment of apoptotic responses [352]. Pharmacological inhibition of both PI3K and BRAF increases BIM levels and potentiates apoptosis [352], suggesting that AKT signaling contributes to resistance of melanomas with activating BRAFv600E mutations to BRAFi (Figure 4). Mutations in the alpha catalytic subunit (PIK3CA) of PI3Ks have also been associated with first-line chemoresistance to folinic acid, fluorouracil, and oxaliplatin (FOLFOX) treatment regimens in cases of colorectal cancers [353]. Treatment of a patient-derived chemoresistant colorectal cancer cell line model with the PI3K inhibitors perifosine or LY294002, reduced tumorigenesis and sensitized resistant cells to FOLFOX both in vitro and in vivo (Figure 4) [353].

Clinical trials investigating the therapeutic benefit of various small molecule inhibitors targeting pathway components of PI3K/AKT signaling are currently underway, and show great promise [349,354,355,356]. Everolimus is a mTORC1 inhibitor that has been FDA approved for use in combination treatment of advanced and/or metastatic cases of breast cancer [357], renal cancer [358], and neuroendocrine tumors [359]. It should also be noted that, through its inhibitory effects on mTORC1, everolimus also induces the autophagy pathway [360]. This suggests that, in certain cancer contexts, autophagy can function as an anti-tumorigenic process. Clinical trials investigating the clinical benefit of everolimus in combination treatment of other cancers are also underway (http://clinicaltrials.gov/).

##### AKT Signaling and Autophagy: Connections to Treatment Resistance in Cancers

**Figure 5:**
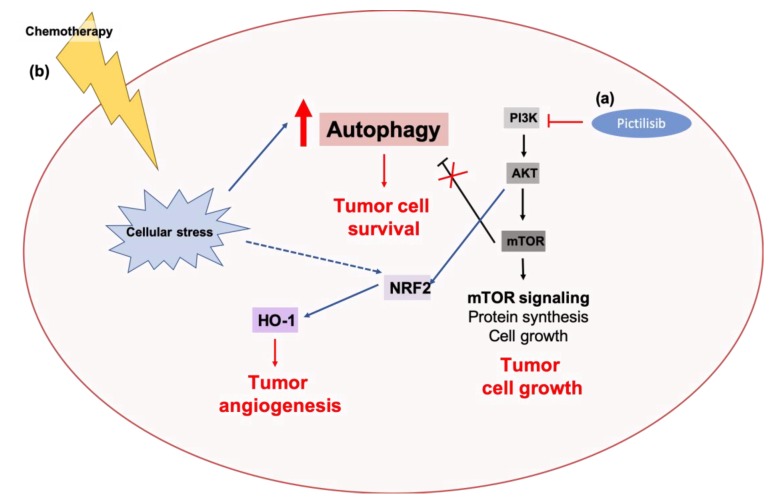
Associations between AKT signaling and autophagy in tumor tolerance against targeted therapies and chemotherapies. (**a**) The pan-PI3K inhibitor, pictilisib, inhibits PI3K-mediated AKT activation, and results in cytoprotective autophagy. Pharmacological and genetic inhibition of autophagy potentiates the pro-apoptotic and growth inhibitory effects of pictilisib in breast cancers [262]. (**b**) Chemotherapy-induced cellular stress results in the activation of autophagy and the cytoprotective enzyme, heme oxygenase-1 (HO-1). HO-1 is transcriptionally upregulated by NRF2, a downstream target of AKT. Pharmacological inhibition of AKT signaling, by PI3K inhibition, potentiates the cytotoxic effects of anthracyclines, like pharmorubicin, and is associated with a reduction of autophagy and HO-1 levels [362].

Associations between the autophagy pathway and AKT signaling have been implicated in the progression and acquisition of treatment resistance in various cancers. Studies in prostate cancer have demonstrated that the essential autophagy gene, Atg7, cooperates with PTEN loss of function aberrations to promote in vivo tumorigenesis [361]. The genetic deletion of Atg7 in mouse models of PTEN-deficient prostate cancer was associated with reduced tumor growth and elevated ER stress, suggesting a crosstalk between the autophagy pathway and hyperactivated AKT signaling in tumorigenesis [361]. It was recently demonstrated that treatment of estrogen receptor-overexpressing (ER+) breast cancer cell lines with the pan-class I PI3K inhibitor pictilisib, induces autophagy as a cytoprotective mechanism [262]. Both pharmacological inhibition of autophagy using the lysosomotropic agent, CQ, or genetic inhibition of autophagy through RNAi-mediated knockdown of ATG5 or ATG7 potentiated the pro-apoptotic and growth inhibitory effects of pictilisib in vitro and in vivo (Figure 5) [262]. Studies in triple-negative breast cancers (TNBC) also demonstrated that both the autophagy pathway and PI3K/AKT signaling contribute to resistance to anthracyclines. Treatment of TNBC cell lines with the anthracycline pharmorubicin, was associated with an increase in levels of autophagy and heme oxygenase-1 (HO-1), a cytoprotective enzyme that catalyzes the oxidation of heme to produce biological molecules, like carbon monoxide (CO), ferrous ions (Fe2+, Fe3+), and biliverdin [362]. Elevated levels of HO-1 have been associated with the growth and angiogenesis of prostate [363,364] and lung cancers [365,366]. It was also demonstrated that PI3K/AKT signaling regulates the expression levels of HO-1 in TNBC at both the mRNA- and protein-level [362]. Pharmacological inhibition of PI3K/AKT signaling with the PI3K inhibitor, LY294002, in combination with pharmorubicin was associated with an additive reduction in cell survival, autophagy, and HO-1 mRNA and protein levels [362]. These studies suggest a correlation between PI3K/AKT signaling and autophagy in the regulation of cytoprotective mechanisms and chemotherapy response (Figure 5). Previous studies have also demonstrated that the inhibition of autophagy, pharmacologically using CQ or genetically by ATG5 or ATG7 knockdown, potentiates the cytotoxic effects of the anthracycline, epirubicin, in TNBC [99]. These studies further suggest a crosstalk between the PI3K/AKT and the autophagy pathway in treatment response in cancers.

To date, the molecular mechanisms underlying how AKT signaling interacts with the autophagy pathway to promote treatment resistance also remains unclear. Clinical trials examining the potential of combined targeting of the autophagy-PI3K/AKT axis in cancer are currently underway. For example, the therapeutic potential of combination treatment with everolimus and HCQ in advanced cases of renal cell carcinoma are currently undergoing mid-phase clinical trials [240]. Similarly, early clinical trials investigating the therapeutic benefit of combination treatment with the AKT inhibitor MK2206, and HCQ in various solid tumors, like melanoma, prostate, and kidney, are currently in progress [347,367]. It is important to note that treatment with everolimus [240,360] or AKT inhibition [367] both inhibit mTORC1 and induce autophagy. The ability of either in augmenting the therapeutic effects of HCQ-mediated autophagy inhibition highlights a complex relationship between AKT signaling and the regulation of autophagy, which remains to be clarified.

### 3.2. The Cellular Recycling and Self-Degradative Functions of Autophagy Contribute to Chemoresistance in Cancers

Despite many pre-clinical and clinical studies supporting the pro-tumorigenic roles of autophagy in various cancers, the molecular mechanisms underlying how autophagy acts to alleviate cytotoxic stresses that stem from anti-cancer treatments remain poorly understood. However, as highlighted in this section, a few studies establishing molecular contributions of autophagy in chemoresistance have recently emerged.

#### 3.2.1. The p62-KEAP1-NRF2 Axis

##### p62-KEAP1-NRF2 Signaling in Cancers

The process of autophagy occurs under both basal conditions and conditions of stress. Stress-induced autophagy is usually equated with selective autophagy, which involves the removal of specific substrates recognized by autophagy cargo receptors [368,369,370]. One such autophagy cargo receptor is the ubiquitin binding protein Sequestosome 1(SQSTM1), also known as p62. p62 is a pleiotropic scaffolding protein that has been implicated in various cellular processes, and dysregulation of p62 function has been implicated in pathological diseases, like cancer [371].

NF-E2-related factor 2 (NRF2) is a major transcription factor that plays important roles in regulating cellular oxidative stress responses. The Kelch-like ECH-associated protein 1 (KEAP1) is an adaptor protein that functions as part of an ubiquitin ligase complex that is involved in cellular proteosomal degradation [371]. Under basal conditions, KEAP1 interacts with NRF2 and targets it for proteosomal degradation, thereby downregulating levels of NRF2 in the cell. The oxidation of KEAP1 under conditions of oxidative stress inhibits the interaction between KEAP1 and NRF2, and this increases the overall cellular levels of active NRF2 in the cell [372]. One major function of NRF2 in oxidative stress response is its transcriptional upregulation of genes containing the antioxidant response element (ARE) in their regulatory regions. Some of these genes encode for metabolic enzymes, stress response proteins, autophagy-related (ATG) proteins, and adaptor proteins like p62 [372].

The p62-NRF2-KEAP1 signaling axis has been implicated in promoting cancer progression through metabolic reprogramming. Studies in hepatocellular carcinoma (HCC) have previously demonstrated that the phosphoactivation of p62 is associated with elevated levels of nuclear NRF2 and an increase in the expression of various NRF2 target genes that are involved in the regulation of metabolic processes [373]. Examples include genes involved in the pentose-phosphate pathway, glutathione biosynthesis, and glutaminolysis [373]. The phosphoactivation of p62 in HCC cell lines was also associated with increased growth rates and resistance to anti-cancer agents, like sorafenib and cisplatin [373]. Similar observations regarding the pro-tumorigenic functions of the p62-NRF2-KEAP1 signaling network have been made in other cancers, like pancreatic [374], ovarian [375], and breast [376].

##### Crosstalk between Autophagy Pathway and the p62-KEAP1-NRF2 Axis in Cancer Progression and Chemoresistance

**Figure 6:**
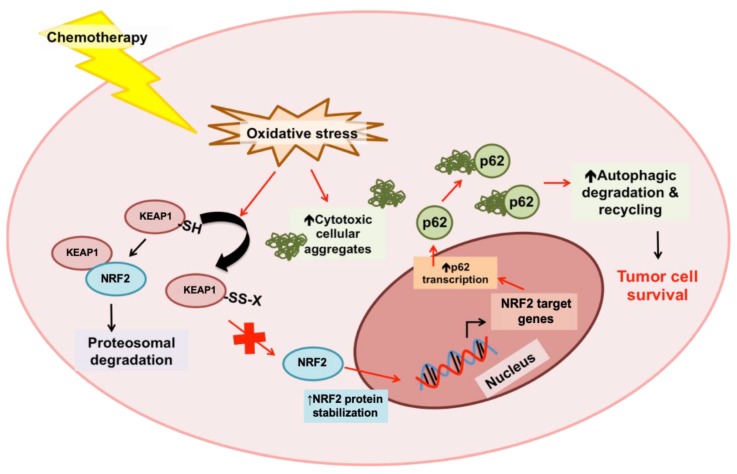
Autophagy mediates the clearance of p62-bound cytotoxic cellular aggregates generated by chemotherapy-induced oxidative stresses. Chemotherapy-induced oxidative stress results in the oxidation of the ubiquitin ligase complex adaptor protein, KEAP1. This inhibits the interaction between KEAP1 and the oxidative stress response transcription factor NRF2, and stabilizes cellular levels of NRF2. NRF2 transcriptionally upregulates various target genes involved in cellular anti-oxidation and stress response, like p62. The autophagy cargo receptor, p62, binds to cellular aggregates generated by chemotherapy-induced oxidative stress, and targets these cytotoxic aggregates for degradation and recycling through the process of autophagy [377].

Recent studies have demonstrated that crosstalk between the autophagy process and the p62-NRF2-KEAP1 signaling axis are involved in mediating tumor tolerance to chemotherapy [377]. An increase in basal oxidative stress and elevated expression levels of NRF2 target genes, including p62, and various ATG genes were observed in epithelial cancer cell lines resistant to cisplatin, docetaxel, and 5-FU [377]. Treatment of resistant lines with chemotherapy was associated with an increase in NRF2 activity, and p62 protein levels and stability [377]. An increase in autophagy-mediated clearance of p62-bound cellular aggregates was observed in chemo-resistant cells, and both the genetic and pharmacological inhibition of autophagy sensitized resistant epithelial cancer cells to chemotherapy [377]. The genetic silencing of p62 in combination with autophagy inhibition was not associated with a further increase in chemosensitivity [377]. p62 silencing alone was associated with a reduction in chemosensitivity, whereas the expression of a truncated p62 mutant lacking ubiquitin- and LC3-binding domains, and the NRF2-activating domain significantly increased chemosensitivity [377], suggesting that the anti-cancer effect of p62 inhibition was autophagy-dependent. These studies support a model whereby KEAP1 is oxidized by chemotherapy-induced oxidative stress, which inhibits interactions between KEAP1 and NRF2 thereby stabilizing levels of the latter in the cell. This consequently results in an upregulation of the NRF2 target gene p62. The p62 adaptor protein binds to cellular aggregates that are generated as a result of chemotherapy-induced oxidative stress, and these aggregates are then degraded and recycled by autophagy. Taken together, this model proposes a functional role of autophagy in mediating the clearance of potentially cytotoxic cellular aggregates generated by chemotherapy, promoting tumor survival and chemoresistance [377] (Figure 6). Most recently, it was also demonstrated that genetic inhibition of autophagy, through ATG7 knockout, is associated with defects in proteolysis and an increase in NRF2 signaling through a p62-KEAP1 mechanism [378]. The genetic inhibition of NRF2 in autophagy-deficient cancers was associated with a greater increase in apoptosis in response to proteasome inhibition by bortezomib, compared to NRF2 inhibition or autophagy inhibition alone [378]. These studies suggest that NRF2 signaling compensates for autophagy defects in cancers, and further supports a crosstalk between the autophagy pathway and NRF2 signaling in contributing to tumor survival and treatment resistance.

#### 3.2.2. The FOXO3A-PUMA Axis

##### FOXO3A-PUMA Signaling in Cancer

Apoptosis is a form of cell death mediated by a distinct intracellular proteolytic cascade, and its dysregulation is often associated with cancer progression and treatment resistance [379,380]. There are two main apoptotic pathways that may be induced depending on the apoptotic signal, as follows: (i) The extrinsic apoptotic pathway, which is induced by the activation of death receptors and (ii) the intrinsic apoptotic pathway, which is induced by intracellular signals in response to cellular stresses, like radiation and chemotherapy [379,380].

Death receptors involved in the extrinsic apoptotic pathway include tumor necrosis factor receptors (TNFRs), TNF receptor superfamily member 6 (TNFRSF6/FAS), and TNF-related apoptosis-inducing ligand receptors (TRAILRs) [379,380]. Death receptor activation results in the recruitment of adaptor proteins, like Fas-associated protein with death domain (FADD) and Tumor necrosis factor receptor type 1-associated DEATH domain (TRADD), that initiate the formation of a death-inducing signaling complex (DISC) by recruiting capase-8 and -10, which are protease enzymes that function as initiators of the apoptotic response. Activation of these initiator caspases is followed by the activation of the downstream effector caspases, caspase-3, -6, and -7, that cleave essential cellular substrates and potentiate cell death [379,380].

The intrinsic apoptotic cascade is induced by intracellular stress signals, like DNA damage, hypoxia, and oxidative stress, that activate members of the B-cell lymphoma 2 (Bcl-2) homology domain 3 (BH3)-only family of Bcl-2 proteins, like BH3 interacting-domain (BID) death agonist and p53 unregulated modulator of apoptosis (PUMA) [379,380]. Pro-apoptotic BH3-only Bcl-2 proteins inhibit the activity of anti-apoptotic proteins, like Bcl-2, B-cell lymphoma-extra large (Bcl-xL), and induced myeloid leukemia cell differentiation protein-1 (Mcl-1). BH3-only Bcl-2 proteins also induce the oligomerization of pro-apoptotic Bcl-2-associated X protein (BAX) and Bcl-2 antagonist or killer (BAK) in the outer mitochondrial membrane, and this results in the formation of supramolecular channels on the mitochondria, also known as mitochondrial outer membrane permeabilization (MOMP). MOMP facilitates the leakage of proteins from the mitochondrial intermembrane space that promotes cell death [379,380]. The regulation of BH-3 only Bcl-2 proteins can occur at the transcriptional level by transcription factors, like forkhead box O3A (FOXO3A) and transformation-related protein 53 (p53) [381,382,383,384].

##### Crosstalk between Autophagy Pathway and the FOXO3A-PUMA Axis in Cancer Progression and Chemoresistance

**Figure 7:**
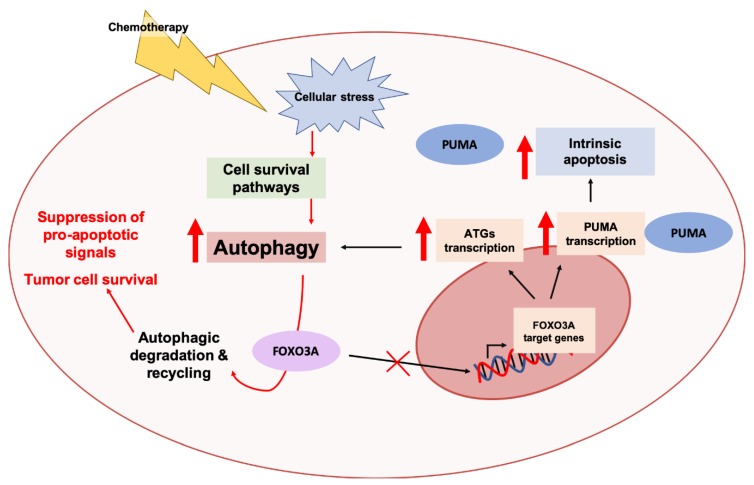
Autophagic degradation of the transcription factor FOXO3A confers tumor tolerance to pro-apoptotic signals. FOXO3A is a transcription factor involved in the transcriptional upregulation of pro-apoptotic genes, like PUMA, and genes involved in cellular stress responses, like autophagy-related genes (ATG). Autophagy is upregulated in response to chemotherapy-induced cellular stresses as a pro-survival response. Stress-induced autophagy mediates the degradation and turnover of FOXO3A, thereby greatly mitigating the transcription of genes encoding pro-apoptotic proteins, like PUMA, and reducing sensitivity to apoptosis [189].

Like autophagy, the apoptotic machinery is a key catabolic process in cells and various nodes of crosstalk between these two processes have been extensively reported in the literature [385,386]. Previous studies in various cancer cell lines have demonstrated that the autophagic program is involved in regulation of the intrinsic apoptotic pathway [385,386]. Autophagy is able to temporally regulate MOMP and cellular responses post-MOMP [385,386]. Induction of autophagy was associated with delays in MOMP, and inhibition of autophagy caused a selective increase in levels of the pro-apoptotic protein PUMA [387]. Similar to autophagy induction, reduction of PUMA levels was associated with a delay in MOMP [387]. Autophagy inhibition sensitized cells to apoptosis, whereas knockdown of PUMA delayed the initiation of apoptosis and rescued cell death associated with autophagy inhibition following apoptotic stimuli [387]. These studies suggest that autophagy may regulate the kinetics and degree of intrinsic apoptosis through regulation of cellular PUMA levels [387]. The underlying mechanisms in regard to how this regulation occurs were recently uncovered using a model of colorectal cancer [189]. Genetic and pharmacological inhibition of autophagy was associated with an increase in PUMA levels at the transcriptional level independent of p53 [189]. However, this increase was not observed when FOXO3A, a transcription factor previously shown to regulate the expression of PUMA [383] and various ATG genes [388,389], was inhibited. An increase in binding of FOXO3A to an intronic response element of the PUMA gene was observed following autophagy inhibition, supporting its role in the regulation of PUMA transcription. It was also demonstrated that protein, and not mRNA, levels of FOXO3A increased following autophagy inhibition. FOXO3A localized to autolysosomes under basal conditions, and accumulated in the nucleus following autophagy inhibition [189]. Autophagy inhibition, in combination with the chemotherapeutic cytotoxic agents etoposide and doxorubicin, was associated with an increase in apoptosis only in colorectal cancer cells that expressed PUMA [189]. Collectively, these studies present a mechanistic model whereby autophagy regulates protein levels of FOXO3A, and, consequently PUMA and apoptosis. Under conditions of chemotherapy-induced stress, autophagy is upregulated and this reduces FOXO3A and PUMA levels, thereby presenting an outlet by which cancer cells evade apoptosis [189] (Figure 7).

## 4. Conclusions and Perspectives

Autophagy has long been considered Janus-faced in the onset and progression of cancers. It is recognized that various nodes of crosstalk between autophagy and many signaling pathways exist, and these nodes can contribute to tumor tolerance against anti-cancer treatments. As such, there has been a longstanding interest in identifying cancer contexts that may benefit from autophagy inhibition in treatment. Previous studies have established key associations between autophagy and major pathways, like MAPK and AKT signaling, that contribute to resistance to both chemo- and targeted therapies in various cancers (Figure 3 and Figure 5). Clinical trials investigating the therapeutic benefit of autophagy inhibition in combination with chemotherapy and/or targeted therapies are currently underway.

Despite these efforts, there still exists a large gap in our knowledge regarding the actual molecular mechanisms underlying how the autophagy process contributes to treatment resistance in cancers. Better understanding of the molecular contributions of autophagy to tumor tolerance against anti-cancer therapies is important for developing rational and more effective combination strategies to improve patient responses to current treatments. To date, two mechanistic models have been proposed that describe how autophagy may act to relieve apoptotic signals and cytotoxic stresses that stem from chemotherapy, thereby promoting tumor survival in treatment settings. Oxidative stress that occurs as a result of chemotherapeutic treatment generates toxic cellular aggregates that promote apoptosis [390,391]. One recent model proposed that how cancers may overcome chemotherapy-induced cell death is through stabilization of the oxidative stress response transcription factor NRF2. NRF2 upregulates the autophagy cargo receptor p62, which binds to cytotoxic cellular aggregates, and consequently delivers them to the autophagy machinery for removal [377] (Figure 6). To overcome pro-apoptotic signals activated in response to cancer treatments, autophagy may also be upregulated to promote the turnover of the pro-apoptotic transcription factor, FOXO3A [189]. This consequently reduces cellular levels of pro-apoptotic proteins, like PUMA, and promotes tumor survival and allows for tumor evasion of apoptosis [189] (Figure 7). Taken together, both these models provide strong rationale for autophagy inhibition as a potential therapeutic strategy in enhancing the cytotoxic effects of chemotherapy.

However, given the dual, context-dependent roles of autophagy in tumor onset and progression, several questions remain, as follows: What cancers are most prone to developing adaptations to circumvent autophagy inhibition strategies [378]? Is there a therapeutic window during which autophagy inhibition will be most beneficial [392,393]? What are the cellular and cancer contexts in which autophagy inhibition will be therapeutically beneficial [22,394,395,396]? Are there indirect or direct caveats to autophagy inhibition in certain cancer contexts, and how can we minimize them [395,397,398]? Are there clinical and/or molecular tumor features that will allow us to better stratify patients that may benefit from autophagy inhibition in treatment [399,400,401,402]? Furthermore, our understanding of how the crosstalk between autophagy and signaling pathways, like MAPK and PI3K/AKT, contributes to treatment resistance remains largely unknown. Understanding these signaling interplays at the molecular level is a prerequisite for identifying combination strategies in which autophagy inhibition will be beneficial in mitigating cancer progression and treatment resistance.

## Figures and Tables

**Table 3:** Examples of pre-clinical studies demonstrating that autophagy and autophagy-related (ATG) genes contribute to cancer resistance to different targeted therapies.

Targeted Agent	Cancer Types	Mode of Autophagy Inhibition		Ref.
		Pharmacological	Genetic	
Monoclonal antibodies				
Bevacizumab (VEGF (vascular endothelial growth factor) inhibitor)	Gliomas, Colorectal cancers, Liver cancers	CQ, HCQ	BECN1 siRNA (small interfering Ribonucleic Acid), ATG7 shRNA (short hairpin Ribonucleic Acid)	[113,192,193,194,195,196]
Trastuzumab (HER2 (human epidermal growth factor receptor 2) inhibitor)	Breast cancers	3-MA, Baf A1	LC3B siRNA, ATG4B siRNA, ATG12 shRNA, ATG4B siRNA	[197,198,199,200]
Cetuximab (EGFR (epidermal growth factor receptor) inhibitor)	Vulvar cancers, Lung cancers, Head and neck cancers	CQ, 3-MA	BECN1 siRNA, ATG7 siRNA	[201,202,203]
Small molecule inhibitors				
Sorafenib (multi-kinase inhibitor—VEGFRs, PDGFRs (platelet-derived growth factor receptor), RAF (Rapidly Accelerated Fibrosarcoma) kinases)	Endometrial cancers, Liver cancers, Kidney cancers, Gliomas, Desmoid tumors	CQ, 3-MA, Baf A1	BECN1 siRNA, BECN1 shRNA, ATG5 siRNA, ATG5 shRNA, miR (microRNA)-375 (ATG7 knockdown)	[204,205,206,207,208,209,210]
Linifanib (multi-kinase inhibitor—VEGFRs and PDFRs)	Liver cancers	CQ, 3-MA	ATG7 siRNA, ATG5 siRNA	[211]
Sunitinib (Multi-kinase inhibitor—PDGFRs, VEGFRs, KIRs (Killer cell immunoglobulin like receptors), FLT-3 (fms like tyrosine kinase-3), CSF-1R (Colony stimulating factor 1 receptor), RET (rearranged during transfection) kinases)	Ovarian cancers, Kidney cancers, Prostate cancers, Pancreatic cancers	Lys05, CQ, 3-MA, Baf A1	ATG7 siRNA, ATG5 siRNA, ATG5 shRNA, LAMP2 shRNA	[212,213,214,215,216]
Gefitinib (EGFR inhibitor)	Breast cancers, Bladder cancers, Skin cancers, Lung cancers, Liver cancers, Pancreatic cancers	3-MA, Baf A1, CQ, HCQ	ATG12 siRNA, BECN1 siRNA, ATG7 siRNA, ATG5 siRNA, VPS34 siRNA, miR-153-3p (ATG5 knockdown)	[217,218,219,220,221,222,223,224,225,226,227,228]
Osimertinib (EGFR inhibitor)	Lung cancers, Breast cancers, Colorectal cancers	Spautin-1, CQ, 3-MA	BECN1 siRNA, ATG5 siRNA	[229,230,231,232,233]
Erlotinib (EGFR inhibitor)	Lung cancers, Glioblastomas, Head and neck cancers	l CQ, 3-MA, Quinacrine	ATG5 siRNA, ATG7 siRNA, BECN1 siRNA, LC3 siRNA	[228,234,235,236,237,238,239]
Everolimus (mTORC1 (mammalian target of rapamycin complex 1) inhibitor)	Blood cancers, Kidney cancers, Bladder cancers, Breast cancer, Neuroendocrine tumors	HCQ, CQ, Baf A1, 3-MA	-	[240,241,242,243,244,245,246,247]
Temsirolimus (mTORC1 inhibitor)	Colorectal cancers, Skin cancers, Kidney cancers	CQ, HCQ	ATG7 shRNA	[248,249,250]
Dactolisib/NVP-BEZ235 (Dual mTOR/Class I PI3K (phosphoinositide 3-kinase) inhibitor)	Kidney cancers, Liver cancers, Blood cancers, Colorectal cancers, Head and neck cancers, Gliomas, Mesotheliomas, Gastric cancers, Rhabdomyosarcomas, Neuroblastomas, Lung cancers	3-MA, CQ, Baf A1, VPS34 inhibitor (VPS34-IN1)	ATG5 siRNA, BECN1 siRNA	[251,252,253,254,255,256,257,258,259,260]
Buparlisib/BKM120 (Pan-Class I PI3K inhibitor)	Lung cancers	CQ	-	[261]
Pictilisib/GDC-0941 (Pan-Class I PI3K inhibitor)	Breast cancers	CQ	ATG5 siRNA, ATG7 siRNA	[262]
Lapatinib (Dual EGFR/HER2 inhibitor)	Breast cancers, Esophageal cancers	CQ, 3-MA	ATG12 shRNA, ATG5 siRNA, BECN1 siRNA	[200,263,264,265]
Afatinib (EGFR/HER2/HER4 inhibitor)	Lung cancers	HCQ, 3-MA	-	[266]
Bortezomib (Proteasome inhibitor)	Breast cancers, Blood cancers, Pancreatic cancers, Cervical cancers, Prostate cancers, Neuroblastomas	3-MA, HCQ, CQ, Baf A1	LC3B siRNA, ATG5 siRNA, P62 shRNA, GABARAPL1 shRNA, BECN1 siRNA	[267,268,269,270,271,272,273,274,275,276]
Carfilzomib (Proteasome inhibitor)	Blood cancers, Neuroblastomas	CQ, HCQ	-	[277,278,279]
Vemurafenib (B-RAF inhibitor)	Brain cancers, Skin cancers, Thyroid cancers, Colorectal cancers	CQ, HCQ, Baf A1, Lys05	ATG5 siRNA, ATG5 shRNA, ATG7 siRNA, ATG13 siRNA	[280,281,282,283,284,285,286]
Trametinib (MEK1/2 (mitogen-activated protein kinase kinase 1/ 2) inhibitor)	Skin cancers, Pancreatic cancers	CQ, HCQ, PIK-III	Inactive dominant-negative ATG4B^C74A^	[287,288]
AKTi-1/2 (Dual AKT 1/2 (Protein kinase B 1/ 2) inhibitor)	Ovarian cancers, Liver cancers, Prostate cancers	CQ, Spautin-1, 3-MA, Baf A1	ATG7 siRNA, ATG5 siRNA, BECN1 siRNA, BECN1 shRNA	[289,290,291]
AZD5363 (AKT 1/2/3 inhibitor)	Prostate cancers	CQ, 3-MA, Baf A1	ATG3 siRNA, ATG7 siRNA	[292]
Tamoxifen (ER (estrogen receptor) inhibitor; anti-estrogenic analog)	Breast cancers	HCQ, Baf A1, 3-MA	ATG7 siRNA, BECN1 siRNA, LAMP3 shRNA, LC3B siRNA, ATG5 siRNA, BECN1 siRNA	[293,294,295,296,297,298,299]
Imatinib (BCR-ABL (breakpoint cluster region-abelson murine leukemia viral oncogene homolog 1) inhibitor)	Blood cancers, Gastrointestinal cancers, Colorectal cancers	Spautin-1, CQ, HCQ, Quinacrine, Vincristine	ATG7 siRNA, ATG12 siRNA, ATG4B shRNA, BECN1 shRNA, ATG5 shRNA, miR-30a (BECN1 and ATG5 knockdown)	[73,300,301,302,303,304,305,306]
HDIL-2 (high-dose interleukin-2 immunotherapy)	Liver cancers	CQ	-	[307,308]
